# Partnering with social service staff to implement pragmatic clinical trials: an interim analysis of implementation strategies

**DOI:** 10.1186/s13063-023-07757-4

**Published:** 2023-11-17

**Authors:** Lisa A. Juckett, Kimberly P. Bernard, Kali S. Thomas

**Affiliations:** 1 School of Health and Rehabilitation Sciences, The Ohio State University, 453 West 10th Avenue, Columbus, OH USA; 2 School of Public Health, Brown University, Providence, RI USA

**Keywords:** Methodology, Evaluation, Stakeholder engagement, Pragmatic clinical trials

## Abstract

**Background:**

With recent growth in the conduct of pragmatic clinical trials, the reliance on frontline staff to contribute to trial-related activities has grown as well. Active partnerships with staff members are often critical to pragmatic trial implementation, but rarely do research teams track and evaluate the specific “implementation strategies” used to support staff’s involvement in trial procedures (e.g., participant recruitment). Accordingly, we adapted implementation science methodologies and conducted an interim analysis of the strategies deployed with social service staff involved in one multi-site pragmatic clinical trial.

**Methods:**

We used a naturalistic, observational study design to characterize strategies our research team deployed with staff during monthly, virtual meetings. Data were drawn from meeting notes and recordings from the trial’s 4-month *Preparation* phase and 8-month *Implementation* phase. Strategies were mapped to the Expert Recommendations for Implementing Change taxonomy and categorized into nine implementation clusters. Survey data were also collected from staff to identify the most useful strategies the research team should deploy when onboarding new staff members in the trial’s second year.

**Results:**

A total of 287 strategies were deployed. Strategies in the *develop stakeholder interrelationships* cluster predominated in both the Preparation (35%) and Implementation (31%) phases, followed by strategies in the *use iterative and evaluative approaches* cluster, though these were more prevalent during trial Preparation (24%) as compared to trial Implementation (18%). When surveyed on strategy usefulness, strategies in the *provide interactive assistance*, *use financial approaches*, and *support staff* clusters were most useful, per staff responses.

**Conclusions:**

While strategies to develop stakeholder interrelationships were used most frequently during trial Preparation and Implementation, program staff perceived strategies that provided technical assistance, supported clinicians, and used financial approaches to be most useful and should be deployed when onboarding new staff members. Research teams are encouraged to adapt and apply implementation strategy tracking methods when partnering with social service staff and deploy practical strategies that support pragmatic trial success given staff needs and preferences.

**Trial registration:**

NCT05357261. May 2, 2022.

**Supplementary Information:**

The online version contains supplementary material available at 10.1186/s13063-023-07757-4.

## Contributions to the literature

- In alignment with the nature of pragmatic trials, trial teams should use practical methods to track and evaluate the strategies that help social service staff implement pragmatic trial procedures, namely participant recruitment and intervention delivery.
- Strategies that are intended to develop stakeholder interrelationships may be most appropriate to deploy during trial the Preparation phase, before participant recruitment and intervention delivery activities begin.
- Strategies designed to provide staff with individualized technical assistance support, financial incentives, and routine reminders may help enhance the success of pragmatic trial implementation, particularly within the social service context.

## Introduction

In 2021, the Patient-Centered Outcomes Research Institute (PCORI) released its guidance on the design and conduct of *pragmatic clinical trials* [1]. Such trials are implemented in real-world settings, include typical patients as participants, and often require research teams to closely partner with frontline staff with non-research backgrounds [2, 3]. Similar to the Pragmatic Trials Collaboratory initiative established by the National Institutes of Health [4], PCORI’s support of pragmatic trials disrupts the funding patterns of federal agencies who have historically invested an estimated $3 billion annually in explanatory studies [5]. Though necessary for establishing the efficacy of a given intervention or understanding why certain phenomena occur, explanatory studies are often conducted in tightly controlled environments and are characterized by highly selective participant eligibility criteria [6, 7]. These stringent parameters can limit an explanatory study’s relevance to providers, patients, and communities, underscoring the importance of pragmatic trials to produce findings that are more readily implementable in real-world health and social service systems [2, 8].

Central to successful pragmatic trial implementation is the involvement of frontline staff members who assist with participant recruitment activities and intervention delivery [9–12]. In prior pragmatic trials, for instance, research teams have partnered with frontline staff to conduct in-depth medical record reviews and identify patients eligible for trial participation [13], share study recruitment fliers and brochures with patients during routine medical appointments [14], explain general information about the trial and its purpose to potential participants [15], and receive training on specific interventions or programs to be implemented in clinical or community settings [16, 17].

Despite frontline staff’s inherent value when implementing pragmatic trials, there remains little guidance for how research teams can plan and deploy *implementation strategies* that support staff in performing trial activities [18–20]. Drawing from the implementation science field, these “strategies” or “drivers” [21–23] are the techniques and methods that help develop staff’s skills and knowledge for completing trial-related tasks. Examples of strategies may include verbally educating staff on eligibility inclusion versus exclusion criteria, providing demonstrations of medical chart reviews to identify eligible participants, training staff on the use of study-specific tools and technologies, or providing written manuals to standardize staff’s delivery of the intervention(s) being tested [24–26]. While implementation strategies have been extensively studied to determine their effect on the uptake of evidence-based practices (e.g., interventions, programs, treatments) [24, 27, 28], only recently have such strategies been proposed to help advance pragmatic trial implementation [29].

Given their recency, these recommendations to use implementation strategies with staff members involved in pragmatic trials have yet to be thoroughly operationalized. Moreover, few trial teams have intentionally adopted procedures to track, monitor, and evaluate the specific strategies deployed with frontline staff who assist with participant recruitment and intervention delivery [9, 30, 31]. Without procedures to track and reflect on their actions, research teams miss crucial opportunities to identify promising strategies — in real-time — for enhancing staff’s active trial involvement. For instance, as trials progress, the needs of staff will likely change [32, 33], warranting the deployment of new or modified strategies that appropriately match these needs (i.e., after the trial has been underway for months, staff may need fewer strategies to understand participant eligibility criteria but more strategies to reward them for completing participant recruitment activities). Additionally, strategies deployed by the research team may be highly valued by some frontline staff members but not by others [34]. As such, routinely and pragmatically assessing the perceived value of these strategies may allow research teams to obtain insight into strategies that should be maintained, revised, or discontinued with existing staff as well as new staff members who need to be onboarded to trial procedures.

For the present study, we adapted previous implementation science methodologies [32, 35] to track and evaluate our own strategies used with frontline staff involved in implementing a multi-site, pragmatic trial in the social service system. Thus, we conducted an interim analysis of strategies deployed with staff who assisted with participant recruitment and intervention delivery in our trial’s first full year. This paper (a) presents our “pragmatic” methods for tracking and monitoring implementation strategies used during the trial’s 4-month *Preparation* phase and 8-month *Implementation* phase, (b) explores variability in the types (e.g., unique versus repeat) of strategies deployed across Preparation and Implementation phases, and (c) presents our evaluation of strategies perceived by frontline staff to be most useful prior to the trial’s onboarding of new staff members. We conclude by reflecting on opportunities to enhance the success of pragmatic trials implemented in the social service setting.

## Methods

The present naturalistic, observational study was implemented in the context of a pragmatic, two-arm, randomized comparative effectiveness trial. The objective of the trial was to compare the health outcomes (primary – days at home; secondary – food insecurity, loneliness, health-related quality of life; exploratory – dietary intake) of food-insecure older adults on waiting lists at Meals on Wheels programs who were randomly assigned to receive one of the two predominant modes of meal delivery for 6 months: (1) one lunch time meal delivered 5 days a week by a volunteer or paid driver who socializes with the client and performs an informal wellness check or (2) 10 frozen meals that are mailed to participants’ homes every 2 weeks. The trial was conducted in partnership with five social service agencies, specifically Meals on Wheels providers, from across the USA. Our methods described below are reported in accordance with the Standards for Reporting Implementation Studies (StaRI) statement [36].

### Social service frontline staff and trial partnership

**Fig. 1:**
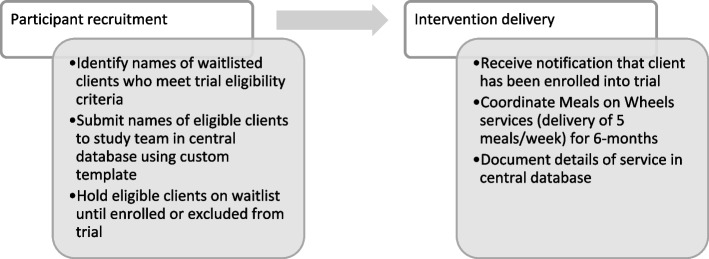
Pragmatic trial activities to be completed by Meals on Wheels staff

Our five Meals on Wheels program partners were located in Florida, California, South Carolina, and Texas whose respective programs reached between 600 and 4500 adults over the age of 60 (Table 1). For the pragmatic trial, frontline staff were tasked with two main activities. First, the research team requested that staff identify individuals from their Meals on Wheels waiting lists who met the trial’s eligibility criteria [37]. Upon confirming eligibility, program staff uploaded the names and sociodemographic characteristics of individuals on waiting lists to the trial’s central database system, via a custom template, to be accessed later by the research team. Second, for enrolled participants who were randomized to receive daily-delivered meals, program staff were also responsible for coordinating daily meal services, which included delivering meals, appropriately invoicing for meals, and documenting changes in participants’ meal preferences. At the end of the 6-month intervention period, programs were tasked with continuing to serve all participants in both arms of the study their usual meal service (Fig. 1).

**Table 1:** Characteristics of Meals on Wheels agencies

	**Number of frontline staff**^**a**^	**Geographical region (USA)**	**Annual number of MOW clients served**
Agency #1	5	West	625
Agency #2	2	Southeast	1000
Agency #3	2	Southeast	650
Agency #4	3	Southwest	4525
Agency #5	2	Southeast	1300

^a^Number of frontline staff = number of staff involved in pragmatic trial activities (i.e., participant recruitment and intervention delivery)

*MOW* Meals on Wheels

### Agency staff team meetings

Frontline staff members across all five agencies participated in monthly, virtual meetings with the research team consisting of the principal investigator, the project director, and 1–2 contracted technology support experts. These monthly team meetings were the only times frontline staff from all agencies convened to formally discuss trial implementation. Accordingly, our implementation strategy tracking efforts were focused on these meetings — an approach that is consistent with prior methods to track implementation strategies in the social service context [35]. During each meeting, the research team updated frontline staff on trial progress and deployed specific strategies to support staff’s involvement in participant recruitment and the coordination of daily-delivered meal services. “Deployed” strategies were those that were mentioned by the research team as having occurred outside of the virtual meeting or were used within the meeting itself. A total of 12 monthly meetings were held from January to December 2022. Each 60-min meeting was structured according to a timed agenda, led by the study’s principal investigator and project director, and recorded via Zoom [38]. In addition to facilitating and recording each meeting, the project director also documented notes directly in the meeting agenda, and notes were shared with agency staff via email 2–3 days after each monthly meeting concluded.

### Strategy coding procedures

#### Detailed strategy descriptions

Upon release of monthly recordings and notes, our study’s implementation specialist reviewed meeting materials by first listening to each meeting recording in its entirety and documenting initial perceptions of the types of strategies used by the research team. The specialist then re-analyzed each recording, paused the recording when a strategy was deployed, and manually documented a detailed description of the strategy. This approach was used for two reasons. First, the initial review of the meeting recording allowed our implementation specialist to develop a general understanding of the strategies deployed, and secondly, by re-reviewing each recording, the specialist was able to thoroughly describe how the research team operationalized each strategy for the trial context. Once all recordings were re-reviewed by the implementation specialist and strategies had been sufficiently documented, the specialist confirmed strategy details by referencing the project director’s meeting notes from the monthly meeting agendas. Lastly, strategies were vetted with the principal investigator and project director to verify the accuracy and completeness of strategy descriptions.

#### ERIC strategy codes

Once all deployed implementation strategies had been documented in detail, our implementation specialist coded each strategy according to terminology from the Expert Recommendations for Implementing Change (ERIC) taxonomy [22] — a catalog of 73 strategies hypothesized to support staff’s skills for implementing new practices or procedures. Our specialist had prior expertise in characterizing strategies using the ERIC taxonomy [24, 39, 40] and also used supplementary materials (e.g., expanded strategy definitions and examples) from the original ERIC taxonomy publication to guide coding procedures. Our decision to use a single coder aligned with key principles of pragmatic trials in that one coder with implementation strategy expertise was more “pragmatic” than dedicating additional resources to train multiple coders to complete the same task in a specified timeframe [41].

#### Strategy clusters

After strategies were coded using ERIC terminology, they were each grouped into one of nine implementation clusters. Clusters were originally conceptualized through concept mapping methods led by Waltz et al. [42]. These nine clusters included the following: (1) develop stakeholder interrelationships, (2) use evaluative and iterative approaches, (3) provide interactive assistance, (4) train and educate staff, (5) adapt and tailor to context, (6) use financial approaches, (7) change infrastructure, (8) support staff, and (9) engage consumers.

#### Time of strategy deployment

Tracked strategies were deployed between January 2022 and December 2022, and our implementation specialist documented the specific month each strategy was used. For the purposes of our trial, we denoted our *Preparation* phase to be the first 4 months (January 2022 – April 2022) before trial participants were actively recruited and prior to any element of intervention delivery. Our *Implementation* phase was defined as the 8-month time frame from May 2022 (when the first participant was enrolled) to December 2022.

#### Repeat and unique strategies

Although our coding methodology was heavily informed by previously established strategy tracking procedures [32, 35], we expanded these procedures by distinguishing “unique” strategies from “repeat” strategies. Unique strategies were those that were deployed only once (i.e., one-off strategies) over the course of our trial’s first 12 months. Repeat strategies were those that the research team used two or more times with frontline staff members. Differentiating repeat strategies from unique strategies also informed the development of our custom survey (described below) to evaluate the perceived usefulness of strategies that were most frequently deployed. All strategy coding data were documented into an Excel (Version 2202) template that consisted of the following data fields: detailed strategy description, ERIC strategy code, strategy cluster, month of strategy deployment, deployment phase (i.e., Preparation or Implementation), and unique versus repeat strategy distinction.

#### Frontline staff survey

Prior to initiating our trial’s second year of participant recruitment and intervention delivery, we aimed to evaluate the usefulness of common strategies deployed as perceived by frontline staff. Our interest in evaluating these strategies was driven by our need to onboard new staff members from three additional Meals on Wheels programs who would be tasked with recruiting participants and delivering the intervention (daily-delivered meal services). Given that new staff members would be onboarded during the Implementation phase of the trial, we sought to evaluate the usefulness of only those strategies deployed during the 8-month Implementation phase. Commonly used strategies, as determined by the implementation specialist, principal investigator, and project director, were compiled into a Qualtrics [43] survey that was informed by the Implementation Strategy Satisfaction Survey [34]. To establish face validity, the survey was piloted by one frontline staff member who was unaffiliated with the present pragmatic trial and one representative from Meals on Wheels America. During the December 2022 virtual meeting, the implementation specialist was allotted time to provide staff with a description of the survey’s development and explained the survey’s intent to identify useful strategies that could be replicated with newly onboarded staff members. All staff who were present for the virtual meeting and had attended at least one prior meeting were invited to complete the electronic survey via an anonymous link. Survey items requested that staff provide the name of the Meals on Wheels program they represented and their rankings (1 = not at all useful; 5 = extremely useful) of 11 commonly deployed strategies used to support staff’s ability to complete participant enrollment and intervention delivery procedures. Staff were provided 5 min during the December meeting to complete the survey and were also sent email reminders 1-day after the meeting, after 2-weeks, and during the next month’s virtual meeting.

### Analysis

Descriptive analyses were used to first calculate frequencies of strategies (ERIC codes and clusters) deployed by the research team across the Preparation and Implementation phases. Based on data fields from our Excel template, we applied univariate techniques to determine the proportion of strategies that were used during each month and in each phase of the trial and also calculated those strategies that were “unique” compared to “repeat.” Survey data collected from frontline staff were also examined by means of univariate analyses to determine the usefulness of strategies commonly deployed. Strategies were considered “highly useful” if 70% of staff rated them as either “very” or “extremely” useful on a 5-point Likert scale. “Less useful” strategies were those that received ratings of “moderately,” “slightly,” or “not at all” useful by at least 25% of staff.

## Results

A total of 287 strategies were deployed across 12 months, representing 24 ERIC strategy codes and all nine implementation clusters. Eighty-eight strategies were used by the trial team during the Preparation phase, and 199 strategies were used during the Implementation phase (see Table 2 for strategy examples). Below, we describe these strategies deployed in each phase, the use of repeat compared to unique strategies, and staff’s perceived usefulness of strategies according to survey data.

**Table 2:** Specific examples of strategies categorized by cluster and ERIC taxonomy definitions

Cluster	ERIC strategy example	ERIC strategy defined^a^	Specific example
**Preparation phase (***n*** = 88)**			
Develop stakeholder interrelationships (*n* = 31)	Conduct local consensus discussions	Include stakeholders in discussions about the importance and appropriateness of an innovation	Gather program input on trial eligibility criteria for waitlisted MOW clients
Use iterative and evaluative approaches (*n* = 21)	Assess for readiness and identify barriers and facilitators	Determine the degree to which an agency is ready to implement an innovation and potential obstacles and strengths to implementation	Invite staff to share their agency’s typical meal service practices (i.e., meal options, number of meals provided)
Provide interactive assistance (*n* = 15)	^b^Share technical information	Deliver technical assistance focused on implementation issues	Review name of study funder, study hypothesis, and study design with all frontline staff
Train and educate staff (*n* = 12)	Distribute educational and preparatory materials	Share manuals, toolkits, and guides in-person, electronically, or by mail	Share PDF documents via email that describe participant eligibility, monthly recruitment targets, and participant information packet
Support staff (*n* = 8)	Remind staff	Develop reminder systems to help staff recall information or use an innovation	Remind staff to enter availability for large-group database training with technology experts
Change infrastructure (*n* = 1)	Change record systems	Modify records to streamline documentation of the innovation	Change the software system through which staff submitted names of waitlisted clients to the study team
**Implementation phase (***n*** = 199)**			
Develop stakeholder interrelationships (*n* = 62)	^c^Cultivate relationships	Foster partnerships with stakeholders involved in the implementation effort	Encourage staff to change names on Zoom to include their respective agency name
Use iterative and evaluative approaches (*n* = 36)	Develop and organize quality monitoring systems	Create procedures that monitor the innovation and the quality of its implementation	Request that all staff call potential participants to confirm contact information before submitting client names to study team
Train and educate staff (*n* = 34)	Conduct ongoing training	Routinely provide training to stakeholders	Hold live Zoom demonstration (with screen-sharing) for how staff should terminate service in database system
Provide interactive assistance (*n* = 31)	Centralize technical assistance	Use a standard system or approach to deliver information about how to best implement the innovation	Appoint technology support experts to respond to questions about documenting start and end date for meal services
Support staff (*n* = 24)	Create new implementation teams	Convene diverse stakeholders to identify approaches to improve implementation of the innovation	Recruit volunteer staff members to participate in workgroup and select preferred formats for enrollment reports
Use financial approaches (*n* = 5)	Alter incentive structures	Identify opportunities to provide rewards for innovation implementation	Hold monthly gift card drawings for agencies who submitted names of waitlisted clients by deadline
Change infrastructure (*n* = 4)	Mandate change	Have leadership declare elements of the innovation that are required to be implemented	Require staff to document which waitlisted clients lived with one another before submitting names to study team
Adapt and tailor to context (*n* = 2)	Promote adaptability	Identify ways an innovation can be tailored to meet local needs while still preserving fidelity	Change monthly due date for waitlisted client names to be submitted to the study team as to not conflict with standard end-of-month tasks
Engage consumers (*n* = 1)	Use mass media	Leverage mailings, websites, social media, television, etc. to spread information	Encourage staff to share trial press kits with their agency administrators, board of director members, and communications teams

*ERIC* Expert Recommendations for Implementing Change

^a^Definitions drawn from Powell et al. [21]. ^b^Referred to as “provide local technical assistance” in the ERIC taxonomy; ^c^referred to as “build a coalition” in the ERIC taxonomy. As originally defined, the term “innovation” may refer to a practice, intervention, or treatment. For our present study, the term “innovation” applies to participant recruitment and intervention delivery procedures

### Strategies deployed by phase

#### Preparation phase

The 88 strategies deployed in the Preparation phase represented the following clusters: *develop stakeholder relationships* (35%), *use iterative and evaluative approaches* (24%), *provide interactive assistance* (17%), *train and educate staff* (14%), *support staff* (9%), and *change infrastructure* (1%). Given that strategies in the *develop stakeholder interrelationships*, *use iterative and evaluative approaches,* and *provide interactive assistance* clusters predominated in the Preparation phase, examples of strategies within these clusters included: *cultivate relationships* among staff members and the research team, *share technical information* about research procedures, *assess for readiness and identify barriers and facilitators*, *obtain and use input from stakeholders*, and *conduct local consensus discussions*.

#### Implementation phase

**Fig. 2:**
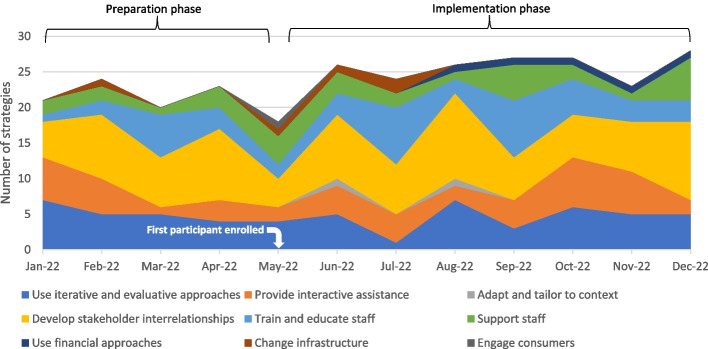
Implementation strategies deployed across the Preparation (Jan 2022–Apr 2022) and Implementation (May 2022–Dec 2022) phases. Visual presentation of strategies adapted from Bunger et al. [32]

Of the 199 strategies used in the Implementation phase, *develop stakeholder interrelationships* (31%) and *use evaluative approaches* clusters predominated (18%), though both of these proportions declined from the Preparation phase. Notably, there was a marginal increase in the proportion of strategies categorized in the *train and educate staff* (17%) as well as *support staff* (12%) clusters. Strategies in the *provide interactive assistance* cluster remained relatively stable (16%) from the Preparation to Implementation phase. Though used less frequently, strategies in the *use financial approaches* (3%), *change infrastructure* (1%), *adapt and tailor to context* (1%), and *engage consumers* (1%) clusters were also deployed in trial Implementation but not during the Preparation phase. Select examples of common strategies used in the Implementation phase included: *remind staff* to complete trial tasks, *conduct ongoing training*, and *distribute educational and preparatory materials*. Figure 2 depicts the proportions of all strategies used, as organized by cluster, across both the Preparation and Implementation phases.

#### Repeat and unique strategies

Within the 287 total strategies deployed with our frontline staff, 223 were considered to be repeat strategies — indicating that three-quarters of all strategies were used two or more times throughout our 12-month analysis period. In the Preparation phase, repeat strategies used by our research team were predominantly categorized in the *develop stakeholder relationships* (30%), *provide interactive assistance* (22%), and *train and educate staff* (20%) clusters. Interestingly, repeat strategies from these same three clusters also predominated in the Implementation phase with relatively stable deployment (30%, 18%, and 20%, respectively).

**Fig. 3:**
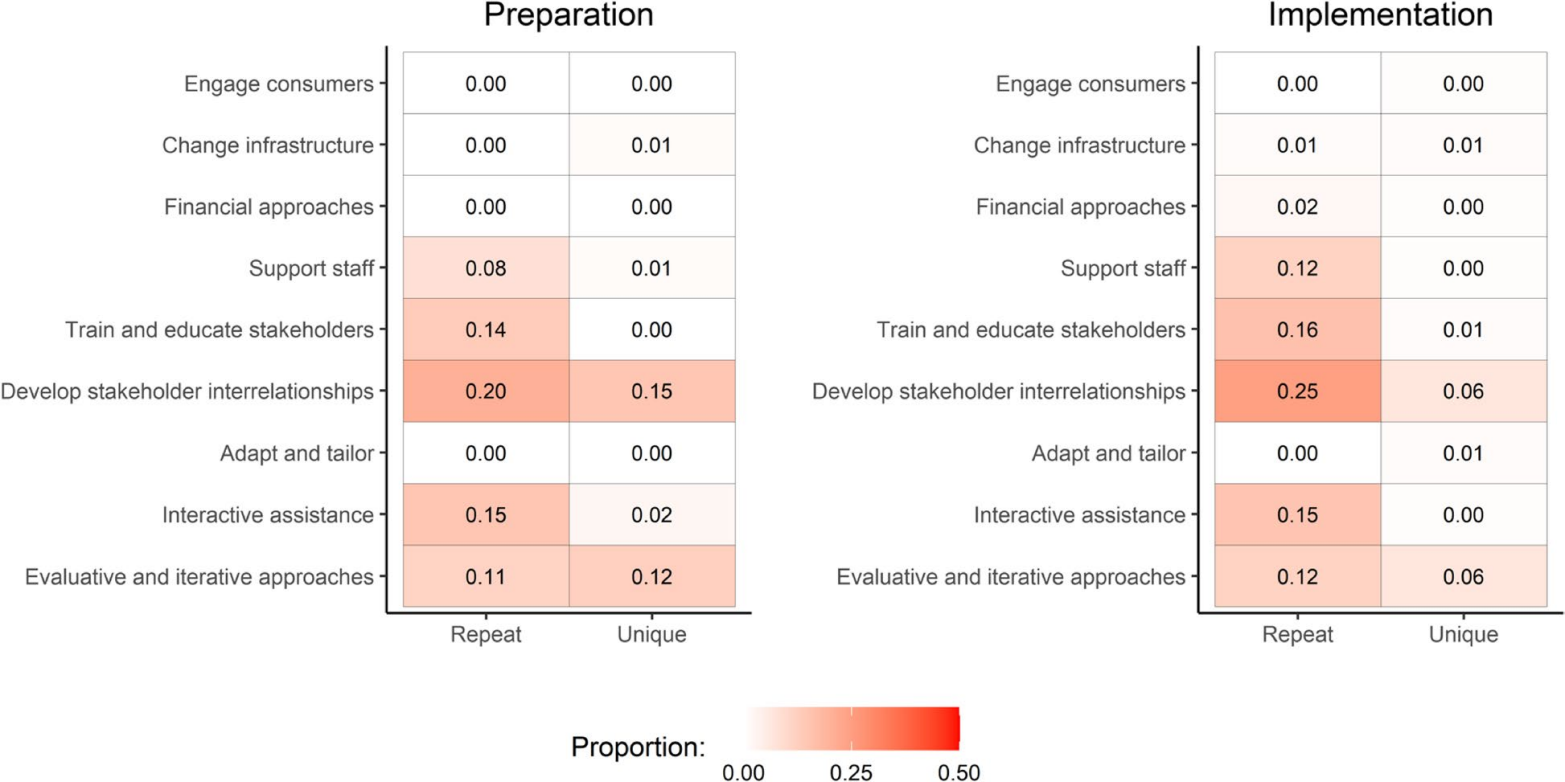
Proportion of repeat and unique strategies across Preparation (January–April 2022) and Implementation (May–December 2022) phases

Though less frequently used, we identified 64 implementation strategies determined to be unique. Of these strategies, 28 (44%) were used in trial Preparation and 36 (56%) during our Implementation phase. In both the Preparation and Implementation phases, strategies in the *develop stakeholder interrelationships* (46% and 36%) and *use evaluative and iterative strategies* (39% and 36%) clusters were most commonly deployed. Figure 3 compares the proportions of unique and repeat strategies used in the Preparation and Implementation phases as categorized by strategy cluster.

### Usefulness of strategies

At least one member from each agency completed our frontline staff survey to evaluate the usefulness of implementation strategies, yielding a 100% response rate across all programs. Thirteen of the 14 staff members invited to complete the survey had been attending monthly virtual meetings since January 2022, indicating low staff turnover throughout the Preparation and Implementation phases.

#### Highly useful strategies

**Fig. 4:**
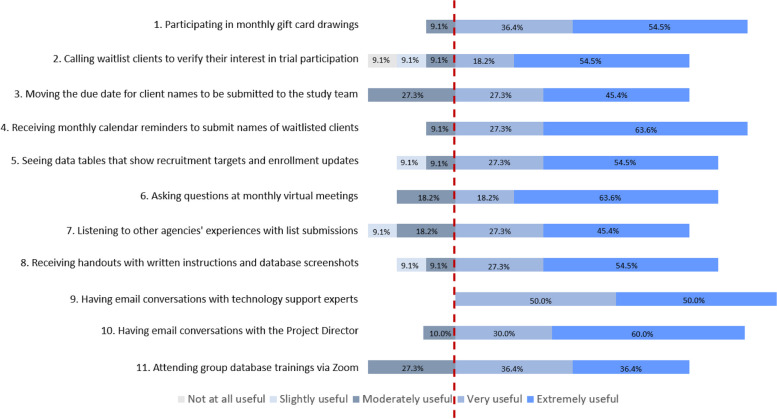
Frontline staff’s perceived usefulness of commonly deployed implementation strategies

Four strategies were rated by staff as being highly useful and were categorized in the *use financial approaches*, *remind staff*, and *provide interactive assistance* clusters. As indicated in Fig. 4, highly useful strategies were as follows: having email conversations with technology support experts, participating in monthly gift card drawings to reward agencies who submitted names of waiting list clients, receiving monthly calendar reminders to submit waiting list client names, and having email conversations with the project director.

#### Less useful strategies

The four less useful strategies, as indicated by ratings of “not at all,” “slightly,” or “moderately” by at least 25% of staff, represented the clusters of *use iterative and evaluative approaches*, *develop stakeholder interrelationships*, *adapt and tailor to context*, and *train and educate staff*. These strategies included calling waiting list clients to verify their interest in study participation, moving the due date for client names to be submitted to the study team, listening to other agencies’ experiences with list submissions, and attending group database trainings via Zoom.

## Discussion

By adapting previously developed strategy tracking methods [32, 35], our interim analysis identified a broad range of strategies to enhance staff’s involvement in our trial’s first 12 months of implementation. In alignment with the pragmatic nature of the present trial, our work showcases practical methods for tracking strategies used to support frontline staff in participant recruitment and intervention delivery activities while also evaluating the usefulness of strategies while the trial was still ongoing. Our practical methods are particularly relevant in our current virtual climate and may be suitable for replication by other trial teams who frequently engage in remote partnerships with frontline staff members.

### Deployment of strategies across the Preparation and Implementation phases

One unexpected finding from our interim analysis was the noticeable shift in the types of strategies that were deployed in the Preparation phase compared to the Implementation phase. Though strategies to develop stakeholder interrelationships predominated in both phases, there was a minor decline in the proportion of these strategies from trial Preparation to Implementation. Notably, this shift is consistent with studies that have similarly tracked strategies to support implementation [32, 44] and indicates that the needs of staff may have changed as the trial progressed. Relatedly, Bunger et al. identified that strategies such as conducting local consensus discussions, obtaining input from staff, and cultivating relationships with frontline staff — or strategies designed to develop stakeholder interrelationships — were deployed more often in the “planning” phase of their multi-component project whereas strategies that provided technical assistance, reminded staff, and conducted audits/provided feedback were used most frequently in the “implementation” phase [32]. The shift in strategies may also reflect the research team’s responsiveness to the barriers and facilitators influencing participant recruitment and intervention delivery. Though the assessment of barriers and facilitators is critical to any implementation effort [45, 46], the Preparation phase allowed the trial team to identify *anticipated* barriers and facilitators to recruitment and intervention delivery whereas the Implementation phase illuminated *actual* barriers and facilitators experienced by frontline staff. In recognition of these actual barriers and facilitators, the research team deployed a greater proportion of strategies to train, educate, and support staff during the Implementation phase.

### Repeat and unique implementation strategies

For our present study, we expanded established implementation strategy tracking methodologies [32, 35] and included procedures to track repeat and unique strategies as well. In prior work, tracked strategies have been reported using the ERIC taxonomy nomenclature (e.g., conduct ongoing training; audit and provide feedback) [32, 34, 35] and implementation clusters (e.g., provide interactive assistance) [44, 47]. While reporting strategies using ERIC codes — as compared to clusters — provides a more robust account of the types of strategies deployed by research teams, it is often difficult to discern strategies used multiple times (i.e., repeat strategies) from one-off strategies (i.e., unique strategies). In the context of pragmatic clinical trials, we emphasize the value of tracking repeat and unique strategies for two reasons. First, despite the growth of the pragmatic trial evidence base, seldom are these trials designed to test the effectiveness of implementation strategies for improving frontline staff’s uptake of a particular intervention or treatment. Rather, strategies used in pragmatic trials are likely deployed in a naturally occurring manner, are rarely operationalized as part of the original pragmatic trial funding proposal, and must be iteratively tailored to meet the needs and abilities of frontline staff members. By routinely (i.e., monthly) tracking repeat and unique strategies, research teams can potentially monitor which strategies are associated with successful trial activities and make necessary modifications to study procedures. Drawing from our own study, for instance, despite using several training sessions to build staff’s skills for identifying eligible trial participants, our research team recognized the need to deploy unique strategies that altered the order in which client names were drawn from program waiting lists and the procedures used to confirm clients’ interest in trial participation. Future analyses could determine the extent to which our repeat and unique strategies were correlated with successful trial activities — namely the achievement of participant recruitment goals — and may be of value to other pragmatic trial teams interested in replicating effective strategies.

Second, tracking repeat and unique implementation strategies is imperative for evaluating their usefulness, particularly when preparing to onboard new staff members to the trial. Staff turnover is an inevitable occurrence in pragmatic trials, and the onboarding of new staff can be time- and labor-intensive for all members of the trial team, especially if the strategies used to build the skills of new staff are not useful or effective. Our identification of repeat and unique strategies informed the development of our frontline staff survey which allowed us to prioritize which strategies, as perceived by staff, were most useful and should continue to be deployed with existing *and* new staff involved in trial procedures.

### Perceived usefulness of strategies

Our survey findings underscored the potential challenges research teams can encounter when attempting to balance strategies that can be generalized to all agencies (i.e., are less time- and resource-intensive) with strategies that meet agency-specific needs and preferences. The most highly useful strategies as perceived by frontline staff were those that involved individualized technical assistance support from the technology support team or the project director. Consistent with prior literature, this finding provides further empirical support for the benefits of technical assistance when staff are introduced to new practices or procedures, particularly when assistance is complemented by tailored training or education [48–50]. Certainly, group training sessions are a rapid and efficient approach to building the skills of frontline staff, but given that our trial’s group training sessions were perceived to be less useful to frontline staff, the deployment of brief, focused technical assistance strategies may serve as a more effective model to optimize the trial-related skills and abilities of staff members [33], particularly given the contextually- and operationally-diverse needs of individual social service agencies [51].

In addition to individualized technical assistance, staff also endorsed the deployment of monthly gift card incentives and calendar reminders to perform trial activities. Beyond their perceived usefulness, routine reminders, such as those automatically generated in electronic documentation systems or delivered via email, have been effective for increasing staff’s completion of specific tasks or implementation of interventions, procedures, or screenings [52]. Moreover, reminders that are delivered at least monthly and are provided in a consistent manner and format, similar to how reminders were delivered by our project director, have led to sustained changes in staff’s job performance and practice behaviors [53, 54]. Gift card incentives have also had favorable effects for promoting staff’s use of new practices and technologies [55], suggesting that “pay-for-performance” incentives may hold great promise for enhancing the implementation of pragmatic trials that rely on the regular involvement of frontline staff members. Pivotal work from Garner et al. established that tiered pay-for-performance rewards (i.e., one reward provided to staff who demonstrated practice competency; a follow-up reward provided if staff implemented practices with high fidelity) were associated with improvements in social service staff’s competence and were also cost-effective and led to desired changes in patient-level outcomes [56, 57]. This work may translate to the pragmatic trial context in that tiered pay-for-performance strategies may first serve to reward individual staff who appropriately identify clients eligible for trial recruitment followed by a second reward provided to staff for each client who chooses to enroll in the trial.

Certainly, financial strategies such as pay-for-performance rewards and gift card incentives may enhance frontline staff’s implementation of trial-related activities, but such strategies — in addition to the customized technical assistance and reminder strategies — are also accompanied by high costs. Though these valued strategies will continue to be deployed in the present trial, our findings illuminate financial and resource limitations that other trial teams should carefully consider when developing their own pragmatic studies. In other words, recognizing the value of staff incentives, individualized technical assistance, and custom reminders early in grant proposal development may help trial teams prepare budget justifications and allocate sufficient funding to cover monetary rewards and the personnel needed to provide ongoing support to staff, including those who need to be onboarded at mid-trial time points.

### Integrating implementation science methodologies into pragmatic trials

Per recent trial conduct recommendations [29], pragmatic clinical trials can be viewed as complex interventions, and trial teams are encouraged to leverage implementation science methodologies — such as strategy tracking — to increase trial success. For the present study, we used a naturalistic, observational approach to track implementation strategies our research team deployed in response to the needs of the trial and the needs of frontline staff. However, the selection and tailoring of our deployed strategies may have been enhanced through the application of implementation theories, models, and frameworks. For instance, during our Preparation phase, our research team made concerted efforts to assess staff’s perceived barriers and facilitators — or determinants — to participant recruitment and intervention delivery procedures. Classifying these determinants using nomenclature from the Consolidated Framework for Implementation Research [58] or the Theoretical Domains Framework [59], as examples, may have informed the systematic selection of implementation strategies that could have been deployed to overcome barriers and capitalize on facilitators. Further, other trial teams may find value in applying additional frameworks such as the Exploration, Preparation, Implementation, and Sustainment framework [60], which can guide the process of planning, initiating, and conducting a pragmatic trial, or evaluation frameworks that can assist trial teams in assessing the extent to which research activities are acceptable, appropriate, and/or feasible to frontline staff members [61].

### Limitations

Though this study makes unique contributions to the pragmatic trial and implementation science bodies of literature, it is not without limitations. First, we recognize that our descriptions of implementation strategies are not fully specified as recommended by Proctor et al. [62]. Though we certainly value these specification recommendations, we claim that our study serves as a foundational, first step towards tracking implementation strategies using practical methods that align with the realistic nature of pragmatic trials. Second, all data sources — meeting notes, meeting recordings, and survey responses — were primarily analyzed by one implementation specialist, potentially threatening the reliability of findings. However, we argue that the analysis of strategies in real-time during ongoing trials should be feasible and assert that the addition of 1–2 secondary coders or reviewers, with similar implementation expertise, would have potentially decreased the efficiency of data analysis, delaying the extent to which our findings could inform strategies deployed when onboarding new staff members. Third, our results only represent the strategies that were mentioned or deployed during monthly virtual meetings. Additional strategies used by the research team with frontline staff were not fully captured, thus, the total frequency of strategies deployed is likely an underestimate. Fourth, although the majority of staff who completed our strategy survey were involved in the trial’s first full 12 months, we were unable to obtain input from staff members who had resigned during the trial’s Implementation phase. Lastly, given technological challenges, strategies deployed during the May 2022 meeting were tracked using meeting minutes only as the meeting could not be recorded in its entirety.

## Conclusion

While our research team used a combination of diverse strategies to support staff in participant recruitment and intervention delivery activities, the most useful strategies included the provision of individualized technical assistance, reminders, and financial incentives. Research teams are encouraged to track implementation strategies deployed in their own pragmatic trials, evaluate strategy usefulness as perceived by frontline staff, and use findings from interim analyses to maximize trial success, particularly for trials in need of onboarding new staff members in the social service setting.

### Supplementary Information

**Additional file 1. **Standards for Reporting Implementation Studies: the StaRI checklist for completion.

**Additional file 2. **CONSORT 2010 checklist of information to include when reporting a randomised trial*.

## Data Availability

The datasets used to track and evaluate implementation strategies from the present study are available from the corresponding author on reasonable request.
